# Visual identifier systems for patients with cognitive impairment in healthcare settings: A survey of practice in UK hospitals

**DOI:** 10.1111/opn.12472

**Published:** 2022-05-16

**Authors:** Karolina Kuberska, Mary Dixon‐Woods, Graham Martin, The DA VINCI Contributor Group, Natalie Armstrong, Jackie Bridges, Tanya V. Brigden, Lesley Carter, Bev Fitzsimons, Joanna Goodrich, Alison E. Hall, Barbara Hodkinson, Chloë Hood, Louise Locock, Alastair S. Macdonald, Alasdair M. J. MacLullich, Colin Mitchell, Sean Ninan, Johan Ordish, Annabel Price, Allyson Rigby, Naomi S. Stockley, Carolyn Tarrant, Benjamin R. Underwood

**Affiliations:** ^1^ THIS Institute (The Healthcare Improvement Studies Institute) University of Cambridge, Cambridge Biomedical Campus, Clifford Allbutt Building Cambridge UK

**Keywords:** cognitive impairment, dementia, hospital care, identification, patient care, person‐centred care, survey

## Abstract

**Background:**

People with dementia and other forms of cognitive impairment form a substantial proportion of patients admitted to hospitals, but problems in their care are persistent. One widely proposed improvement approach involves the use of systems using visual identifiers to help staff quickly recognise people with suspected dementia, with the goal of supporting more personalised care. The aim of this paper is to identify the identifier systems in use and staff perceptions of their strengths and weaknesses.

**Methods:**

We undertook an online survey of staff providing care for people with dementia in acute and mental health hospitals across the United Kingdom. The questionnaire covered different types of visual identifier systems for dementia. It used categorical and open‐response questions to access staff views of their use in practice. Responses were analysed using descriptive statistics, and the Framework approach for free‐text answers.

**Results:**

162 responses were received from staff in at least 48 hospitals. Of these, 128 had direct experience of using visual identifier systems. They reported that multiple identifier systems are in use, including schemes with national scope and locally developed approaches. Most respondents reported that more than one system is in use in their hospital. Different types of identifier were seen to have different strengths and weaknesses. Respondents had a broadly positive view of identifiers, but highlighted risks including lack of reliable and consistent use (linked to competing pressures on staff time), lack of staff training, uncertainty about patient and family views, and unclear consent processes.

**Conclusions:**

Our study suggests that a wide range of identifier systems is in use in UK hospitals, with many hospitals using more than one. Further consideration should be given to ensuring that multiple perspectives—including those of patients and carers—are drawn on in optimising their design, resolving ethical issues and supporting implementation.

**Summary statement of implications for practice**

**What does this research add to existing knowledge in gerontology?**

- Multiple visual identification systems are used for hospitalised people with dementia, with many hospitals using combinations of two or more systems.
- Staff views of visual identifiers for patients with cognitive impairment are broadly positive, but they also highlight risks such as inconsistency, lack of staff training and unclear consent processes.

**What are the implications of this new knowledge for nursing care with older people?**

- Good practice in using visual identifiers for patients with cognitive impairment includes considering the wishes of patients and their carers and a clear consent process.
- Nurses and others involved in care of people with dementia should be aware of the different strengths and weaknesses of different systems, and of potential challenges in using multiple systems together.

**How could the findings be used to influence policy or practice or research or education?**

- Policy and practice around visual identification for patients with cognitive impairment should focus on development of a sound evidence base that addresses effectiveness, cost‐effectiveness, training, patient and carer engagement, and ethics.
- An important focus for improvement will be on increasing the interoperability of different identifier systems, but further research into the advantages and disadvantages of standardisation would be of benefit.

## 1 INTRODUCTION

A large proportion of hospital inpatients are affected by cognitive impairment, including both episodic forms (e.g. delirium) and more long‐term impairment arising from progressive neurodegenerative disease (e.g. dementia) (Mukadam & Sampson, 2011). Worldwide, estimates of the prevalence of dementia in acute hospital populations range from 15 to 42 per cent (Bray et al., 2015; Jackson et al., 2017). To avoid harm and distress, it is important that the distinctive needs of this group—for example relating to nutrition, hydration and toilet use, and understanding and consenting to treatment—are met (Jackson et al., 2017; Røsvik & Rokstad, 2020; Scerri et al., 2020; Sillner et al., 2019). However, it may not always be straightforward for those caring for people in hospital settings to recognise readily which patients are living with cognitive impairment (Hermann et al., 2015; Sillner et al., 2019).

One way of addressing this challenge involves use of systems of visual indicators or identifiers to help clinicians and others involved in patient care. The Royal College of Psychiatrists, for example, recommends that all hospitals in the United Kingdom (UK) have some form of identification system in place (Royal College of Psychiatrists, 2017). Identifier systems for people with confirmed and suspected dementia are now in use in many hospitals in the UK. The systems are various and include stickers, wristbands and notices placed at the bedside or on electronic whiteboards on wards. The purpose of such identifiers is to serve as a visual reminder to staff that a patient may have additional needs, perhaps unrelated to the reason for hospitalisation. Some act as standalone identifiers of cognitive impairment, while others—such as the Butterfly Scheme and the Dementia Friendly approach (Williams, 2011)—are components in wider systems and philosophies of care.

The National Audit of Dementia Care, run by the Royal College of Psychiatrists, reports that as of 2019, 93% of general hospitals in England and Wales use a visual identification system of some description in their wards (Royal College of Psychiatrists, 2019), compared to just 49% in 2013 (Royal College of Psychiatrists, 2017). The National Audit asks only whether a system is in place, and so little is known of which systems are used most frequently across hospitals or how well they work. None of the existing schemes has been subject to full evaluation. Variation in approaches to identification and care of people with dementia or other forms of cognitive impairment may introduce its own risks and burdens, particularly, though not only, when staff and patients move between healthcare organisations (Dixon‐Woods, 2019).

A further challenge is the risks that may arise from widespread use of ‘technologies of attention’, such as visual indicators, particularly in pressured environments (Featherstone et al., 2020). One possible risk is that visual indicators might ironically result in less personalised care, acting as a shorthand for a set of needs to be addressed by routinized responses rather than the preferences and priorities of an individual patient (Featherstone et al., 2020). Use of visual identifiers for confirmed or suspected diagnoses of dementia might also present ethical and legal challenges, for example in relation to obtaining consent from the patient or their advocate, or inadvertent disclosure of personal information to third parties (Brigden et al., 2020). While guidance accompanying some schemes addresses issues of consent and confidentiality (Williams, 2011), there is currently little evidence regarding how such issues are approached in practice.

### 1.1 Aim of the study

The aim of this study was to conduct a survey to map current use of identification systems for patients with confirmed or suspected dementia in organisations in the UK National Health Service (NHS) and to obtain staff views on the systems in use, including their advantages, disadvantages, benefits and risks. The survey was part of a wider study examining the use and design of visual identification systems for people with dementia and other forms of cognitive impairment in institutional settings.

## 2 MATERIALS AND METHODS

We developed an online questionnaire in consultation with an expert collaborative group of 20 people, including staff, patient and carer representatives, individuals who had led the development of existing identification and dementia care systems, clinical and non‐clinical academic experts in related fields, third‐sector organisations, and collaborators in the wider study. We surveyed staff in acute and mental health hospitals in the UK NHS working in areas where adults with confirmed or suspected dementia are assessed or treated.

The questionnaire included a mixture of categorical and open‐ended questions about respondents' opinions regarding visual identification systems, their benefits and their risks, including five‐point Likert scale‐based questions about various characteristics of the systems in use (Supplementary Material 1). It posed questions about nine existing systems that we had identified as being in use nationally through a scoping review, the expert collaborative group and web searches. The questionnaire also invited participants to provide details of other systems, including locally developed ones, that might be in use. In line with established questionnaire‐design principles (Abramson & Abramson, 2008; Streiner et al., 2014), it was user‐tested by members of the expert collaborative group and other volunteers to ensure clarity of wording and consistency of understanding, but it was not formally piloted. The protocol and questionnaire were externally peer‐reviewed and were given ethical approval by the University of Cambridge Psychology Ethics Committee on 6 April 2020. Approval to conduct the research in NHS organisations was provided by the Health Research Authority on 27 July 2020.

The questionnaire was administered using Qualtrics survey software hosted on the Thiscovery (www.thiscovery.org) platform developed by The Healthcare Improvement Studies Institute at the University of Cambridge, where the participants had to register to take part in the study. Given the wide range of clinical and non‐clinical professionals involved in care provision, we sought to include staff of any grade and any occupational group working an NHS hospital with direct involvement in caring for inpatients with dementia. Exclusion criteria covered those who: were not NHS staff, did not have first‐hand experience in caring for adult inpatients with dementia, were under 18, or were unable or unwilling to consent to register with Thiscovery or to participate in the study. All participants were provided with written information about the study and assured that responses were confidential and that neither they nor their employing organisation would be named in outputs. They were asked to confirm their eligibility and give consent to participate at the point of accessing the questionnaire.

Due to the exploratory nature of the study, uncertainty over the size of the population of interest and lack of a reliable sampling frame, we did not specify a target sample size. Our approach to recruitment was instead to seek to secure a diverse sample that might be broadly representative of practice in different types of organisation nationally. Participants were recruited using four parallel approaches. First, research and development directorates of participating NHS organisations were provided with publicity materials, including a poster and a letter of invitation, which they were asked to distribute to key individuals involved in the delivery of dementia care and other relevant areas. Second, these materials were also circulated through the Dementias and Neurodegeneration specialty group of the Clinical Research Network in England, which has strong reach to clinicians in relevant areas with an interest in contributing towards research. Third, details of the study were advertised on social media. Fourth, participants were asked to pass on details of the study to potentially interested colleagues (an approach sometimes referred to as ‘snowball sampling’).

We used descriptive and analytical statistical approaches to analyse responses to categorical questions, and thematic qualitative analysis of responses to open‐ended questions, as set out prospectively in our protocol. We produced summary descriptive statistics relating to profile of respondents; frequency with which use of different systems was reported by participants; processes for deciding to use an identifier for a particular patient; and advantages and disadvantages reported. We used Microsoft Excel to support our quantitative and qualitative analyses.

We planned to use analytical statistics to compare respondents' views of the nine different systems we had pre‐identified. These systems fell broadly into four different *types* (systems using stickers placed near the patient's bed and/or on patient notes; those using wristbands worn by the patient; those using electronic whiteboards and similar technologies away from the patient; and those using summary information kept at the patient's bedside). Since the number of responses received for some individual systems was low, we used analytical statistics (chi‐squared tests, with a predefined significance threshold of *p* < .05) only to compare types of system.

On the advice of the expert collaborative group, though not specified in the protocol, we additionally compared respondents' views on the systems pre‐identified in the questionnaire with their views on locally developed schemes that were similar but not identical to those systems. Answers to open‐ended questions were analysed thematically using methods based on the Framework approach (Ritchie & Spencer, 1994). One researcher read and re‐read open‐ended responses, and developed a coding frame that combined codes derived from the questions with codes generated inductively from the responses. Responses were coded accordingly, and coded summaries reviewed alongside the quantitative results to identify key cross‐cutting themes and complementary, contradictory or mutually explanatory findings.

## 3 RESULTS

The survey ran from October 2020 to January 2021, receiving 183 responses, of which 162 met the eligibility criteria. Respondents were asked to optionally identify their employing organisation. Thirty‐one did not give a response to this question; the remaining 131 participants gave 48 different employing NHS organisations. Eighteen participants came from one NHS organisation; other organisations provided between one and eight participants each (mode 1; median 3). Respondents came from a range of occupational backgrounds (Table 1). The biggest single group was nursing staff (*n* = 59/162, 36%), likely reflecting the predominant responsibility for day‐to‐day care of patients with dementia and other forms of cognitive impairment, including suspected dementia. Allied health professionals (a group that includes physiotherapists, occupational therapists and other professional groups such as podiatrists) (*n* = 28, 17%) and doctors (*n* = 18, 11%) were also reasonably well represented among the respondents. There was a very limited response from non‐clinical staff.

**TABLE 1:** Responses to the question ‘Which option best describes your role in the hospital?’

Professional background	Frequency (percentage)
Doctor	18 (11%)
Nurse	59 (36%)
Nursing / healthcare assistant	9 (6%)
Physiotherapist	6 (4%)
Occupational therapist	10 (6%)
Other allied health professional	12 (7%)
Administrative staff	4 (2%)
Manager	3 (2%)
Other	11 (7%)
No response	30 (19%)
Total	**162 (100%)**

Of the 162 responses included in the final dataset, 34 (21%) stated that their hospital did not use visual identifiers, and were accordingly routed to a truncated version of the questionnaire. Questions regarding the systems used, the approach to administering them and their strengths and weaknesses were addressed to the remaining 128 participants, though response rates to individual questions varied and typically fell slightly short of this figure.

### 3.1 Systems used and decisions about use

We asked participants which system or systems were used in their hospitals, including established national schemes and locally developed schemes. There were four broad types of system: systems using stickers placed near the patient's bed and/or on patient notes; those using wristbands worn by the patient; those using electronic whiteboards and similar technologies away from the patient; and those using summary information kept at the patient's bedside (Table 2).

**TABLE 2:** Responses to the question ‘Which visual identification symbols and systems are used in your hospital?’ respondents could select more than one system

System or symbol	Frequency
Stickers	
The Butterfly Scheme	22
The Forget‐me‐not Scheme	38
Dementia Friendly	48
The Purple Angel	4
Other sticker‐based approach	40
Total stickers	**152**
Wristbands	
Blue wristband	11
Cut‐out symbol in wristband	6
Digital wristband	2
Other wristband‐based approach	17
Total wristbands	**36**
Whiteboards	
Electronic whiteboard	47
Other whiteboard‐based approach	12
Total whiteboards	**59**
Bedside information documents	
‘This is me’ leaflet	59
Other bedside information document‐based approach	18
Total bedside information documents	**77**

**FIGURE 1:**
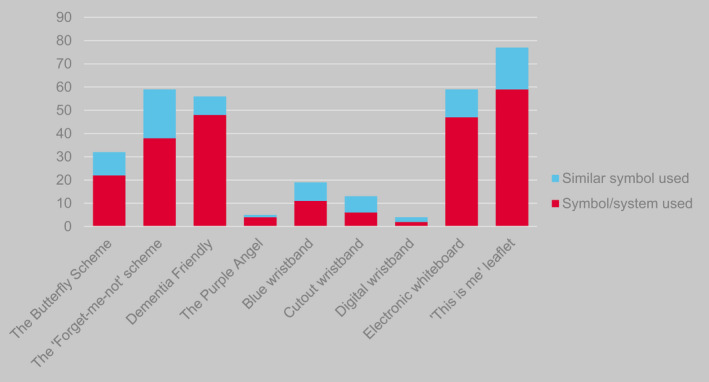
Responses to the question ‘Which visual identification symbols and systems are used in your hospital?’, including those identifying a national scheme (red bar) and those identifying a similar but not identical scheme used locally

The most commonly used systems reported by respondents were national ones. ‘This is me’ booklets were most frequently reported (*n* = 59/121, 49%). This system records brief personal details of patients to facilitate more person‐centred care for those who may struggle to communicate their preferences themselves, for example due to patient's diagnosis of dementia; the booklet's cover serves as a discreet reminder of the patient's diagnosis. The Dementia Friendly scheme was used by 40% of respondents (*n* = 48/121). It involves a flower symbol to identify people affected by dementia. Electronic whiteboards that included information on cognitive impairment were reported by 39% (*n* = 47/121). The Forget‐me‐not Scheme, a wider system of care and training approach that includes use of a sticker featuring a blue flower placed by the patient's bed and/or on patient notes, was reported by 31% of respondents (*n* = 38/121). Participants also reported using systems that were similar but not identical to national schemes, most notably local systems that were similar to the Forget‐me‐not Scheme (*n* = 21/121, 17%) (Figure 1). Free‐text responses confirmed that the most common local variation was an alternative image of a forget‐me‐not flower.

More than one system was in use in the majority of organisations. Over a third (36%) of respondents reported that more than one sticker‐based system was in place in their hospital; 10 respondents reported three systems; one respondent reported that four different sticker‐based systems co‐existed in their hospital. It was also common for different types of systems to be used together. For example, 66 respondents (52%) reported that their hospitals used at least one sticker‐based system alongside at least one summary bedside information system; 51 respondents (40%) reported use of sticker‐based and whiteboard‐based systems. Fourteen respondents (11%) reported that at least one system of each of the four types—stickers, wristbands, whiteboards and summary information sheets—was in use in their hospital.

**FIGURE 2:**
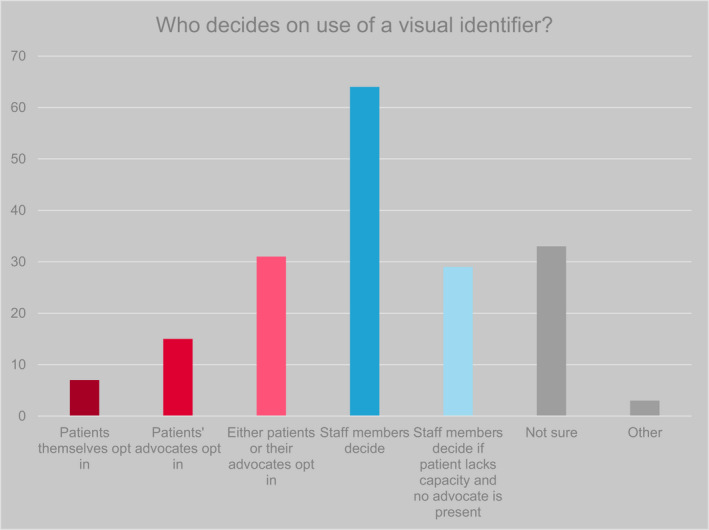
Responses to the question ‘Who chooses whether to use an identification symbol?’ Respondents could select more than one answer

By definition, visual identifiers communicate personal information about individuals to others, including but potentially not limited to staff involved in the individual's care. Ethical concerns, most notably in relation to consent to disclosure, may therefore arise, further complicated by the (potentially fluctuating) capacity of individuals with cognitive impairment to give consent to the user of identifiers (Brigden et al., 2020). We asked respondents who are involved in the decisions to use an identifier for individual patients, including the patient, advocate (i.e. informal [family] carer) and healthcare professional (Table 3; Figure 2). Half of respondents (*n* = 64/127, 50%) indicated that the decision is taken by a healthcare professional; in approximately a third of cases (*n* = 44/127, 35%), this was apparently without input from patients or informal carers.

**TABLE 3:** Responses to the question ‘Who chooses whether to use an identification symbol?’ respondents could select more than one answer

Response	Frequency
Patients themselves opt in	7
Patients' advocates (e.g. relatives or informal carers) opt in	15
Either patients or their advocates can opt in	31
Staff members decide whether to use it	64
Staff members decide only if patient lacks capacity and advocate is absent	29
Not sure	33
Other	3

### 3.2 Strengths, weaknesses, benefits and risks of identification

**FIGURE 3:**
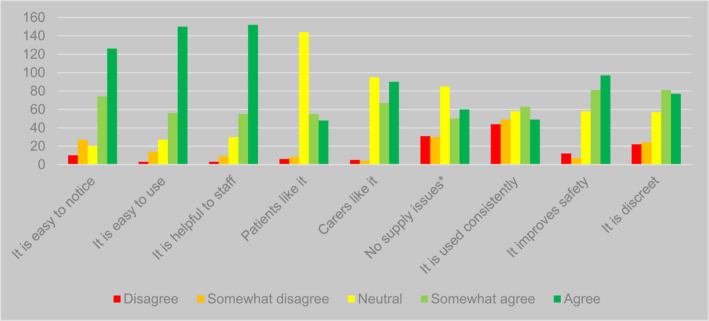
Responses to propositions regarding advantages and disadvantages of identifier systems, aggregated for all systems. *Phrasing of this proposition varied. For most identifiers, it was phrased: ‘We never run out of [identifier].’ For cut‐out symbols in wristbands, it was phrased: ‘It is easy to find the shape cutter when needed.’ For whiteboards, it was phrased: ‘Our electronic board has a special option for patients with dementia’

For each system they reported using, respondents were asked to indicate the extent of their agreement with nine propositions covering issues such as ease of use, impact on quality of care, and views of staff, patients and carers. Table 4 and Figure 3 present the aggregate responses to these questions across all systems in use. Responses were largely positive about key functions of the identifiers: they were seen as easy to notice (*n* = 200/263, 76%, agreed or somewhat agreed), as helpful to staff (*n* = 219/264, 83%), and as benefitting patient safety (*n* = 179/262, 68%), as well as being easy‐to‐use (*n* = 217/263, 83%). Perhaps reflecting uncertainty about the views of patients, 62% (*n* = 162/263) neither agreed nor disagreed with the statement ‘patients like [identifier]’. There appeared to be less uncertainty about a similar statement for carers, with 40% of responses (*n* = 105/264) indicating ‘neither agree nor disagree’. Responses suggested some challenges in implementation of identifier systems. Some related to supply chains, with a quarter (*n* = 64/263, 24%) disagreeing or somewhat disagreeing that there were no supply issues. Forty‐one per cent (*n* = 108/264) agreed or somewhat agreed that identifiers were used consistently.

**TABLE 4:** Responses to propositions regarding advantages and disadvantages of identifier systems, aggregated for all systems

Proposition	Disagree	Somewhat disagree	Neutral	Somewhat agree	Agree
It is easy to notice	12 (5%)	28 (11%)	23 (9%)	76 (29%)	124 (47%)
It is easy to use	4 (2%)	14 (5%)	28 (11%)	62 (24%)	155 (59%)
It is helpful to staff	3 (1%)	10 (4%)	32 (12%)	58 (22%)	161 (61%)
Patients like it	8 (3%)	6 (2%)	162 (62%)	40 (15%)	47 (18%)
Carers like it	6 (2%)	3 (1%)	105 (40%)	68 (26%)	82 (31%)
No supply issues	33 (13%)	31 (12%)	90 (34%)	47 (18%)	62 (24%)
It is used consistently	47 (18%)	54 (20%)	55 (21%)	66 (25%)	42 (16%)
It improves safety	12 (5%)	7 (3%)	64 (24%)	87 (33%)	92 (35%)
It is discreet	22 (8%)	24 (9%)	57 (22%)	81 (31%)	77 (30%)

The free‐text answers offered additional insight into staff views on the benefits and drawbacks of identifiers. Broadly, free‐text responses from those who reported that their hospitals did not currently use visual identification systems tended to be enthusiastic about their potential.

I think they have the potential to be very useful if an appropriate symbol is used and training is disseminated throughout the hospital staff, clinicians and non‐clinicians alike. (Consultant)

Respondents who worked in settings where identifiers were routinely used offered several examples of their advantages. For example, they suggested that visual identifiers such as stickers over the patient's bed or different coloured wristbands could be particularly helpful for staff who were not a regular part of the care team (e.g. locums and bank staff) in identifying patients with potential additional needs. More broadly, visual identifiers were seen as having a role in supporting staff in tailoring their approach to patients with dementia, for example by introducing themselves more explicitly to the patient or allowing extra time as needed.

I work in the elderly medicine admissions ward. Patients change quickly and these visual cues help when the patient asks the staff for the date, the time or help. Often I will be in the room with a patient and another one will ask me to help them because they feel lost. Knowing that they have dementia helps me understand the underlying cause and I can reorient them and reassure them better, as well as providing care in a way that will help reduce their distress and promote their independence. (Nurse)

Wristbands worn by patients themselves with adjustments to indicate a diagnosis of dementia (e.g. blue wristbands instead of white, or wristbands with clasps in a different colour) were considered especially conducive to appropriate person‐centred care. In contrast to other identifiers, wristbands remained with the patient when they moved to other areas of the hospital beyond the ward in which they were staying, for example when being transferred, taken for scans, or leaving the ward unattended.

Patients being transferred from the ward to radiology for a scan who wear a wristband are more easily identifiable as someone who needs more time or explanation or assistance when in an unfamiliar environment, such an X‐ray. Radiology staff are alerted the person may need additional support and should not be left alone when waiting to be collected by the porters. (Physiotherapist)

At the same time, many respondents raised concerns about identifiers. These concerns included the risk that identifiers might, ironically, blur distinctiveness of needs and capabilities and lead to assumptions about the homogeneity of people who had been assigned identifiers, contributing to risks of stigma.

Staff not understanding that visual identifiers are indicators of cognitive impairment or need for adjustments [rather than] a reflection or generalisation of a diagnosis. I have not witnessed this but would be concerned that staff without understanding or training may make assumptions regarding capacity decisions. (Nurse)

Challenges of ensuring consistent use of indicators were reported by 14 respondents. Stickers seemed to be well understood, but were prone to 'coming unstuck' from the individual to whom they were intended to relate. Bedside information documents were seen as containing important information that could inform person‐centred care, but were apt to be ignored when staff were busy. Respondents identified situations where stickers placed above the bed were not removed after discharge, creating the risk that staff would assume that the next patient in that bed also had a dementia diagnosis.

If the previous occupant had been moved but the identifier was still over the bed when the next patient arrived, there may be confusion for staff who take a different ‘tone’with the new patient. (Administrative staff)

Inconsistency was seen to reduce the usefulness of systems, as staff were left less confident that the presence of an identifier accurately reflected a patient's diagnosis or care needs. Equally, they were wary of assuming that patients without identifiers did not have additional needs.

The identifier is not consistently used in ED; this could lead to staff assuming a patient does not have dementia and therefore not treating them accordingly. (Allied health professional)

Reflecting the quantitative finding that staff were less certain of patients' views, some open‐text responses indicated concerns about the acceptability of identifiers for patients or the possible anxieties that use of indicators may cause for patients and carers.

When patients recognise fellow patients do not have the same‐coloured wrist band and asking why. (Occupational therapist)

### 3.3 Strengths and weaknesses of different approaches to identification

We compared respondents' views on the strengths and weaknesses of established national schemes with their views on locally developed schemes (Table 5; Supplementary Material 2, Table 1). For eight of the nine propositions, there was no statistically significant difference in the distribution of views on nationally established versus locally developed schemes; participants were more likely to agree with the proposition ‘Carers like it’ for nationally established schemes than for locally developed ones (though more likely to somewhat agree for locally developed schemes) (*p* = .015).

**TABLE 5:** Responses to propositions regarding advantages and disadvantages of identifier systems, by established national versus locally developed schemes

Proposition	Disagree	Somewhat disagree	Neutral	Somewhat agree	Agree
It is easy to notice					
National schemes	10 (5%)	24 (12%)	15 (7%)	60 (30%)	92 (46%)
Local schemes	2 (3%)	4 (6%)	8 (13%)	16 (26%)	32 (52%)
It is easy to use					
National schemes	3 (2%)	13 (7%)	24 (12%)	49 (25%)	111 (56%)
Local schemes	1 (2%)	1 (2%)	4 (6%)	13 (21%)	44 (70%)
It is helpful to staff					
National schemes	2 (1%)	8 (4%)	28 (14%)	46 (23%)	117 (58%)
Local schemes	1 (2%)	2 (3%)	4 (6%)	12 (19%)	44 (70%)
Patients like it					
National schemes	5 (3%)	6 (3%)	118 (59%)	32 (16%)	39 (20%)
Local schemes	3 (5%)	0 (0%)	44 (70%)	8 (13%)	8 (13%)
Carers like it					
National schemes	4 (2%)	3 (1%)	83 (41%)	42 (21%)	69 (34%)
Local schemes	2 (3%)	0 (0%)	22 (35%)	26 (41%)	13 (21%)
No supply issues					
National schemes	25 (13%)	19 (10%)	73 (37%)	35 (18%)	48 (24%)
Local schemes	8 (13%)	12 (19%)	17 (27%)	12 (19%)	14 (22%)
It is used consistently					
National schemes	36 (18%)	35 (17%)	45 (22%)	50 (25%)	35 (17%)
Local schemes	11 (17%)	19 (30%)	10 (16%)	16 (25%)	7 (11%)
It improves safety					
National schemes	12 (6%)	5 (3%)	49 (25%)	61 (31%)	72 (36%)
Local schemes	0 (0%)	2 (3%)	15 (24%)	26 (41%)	20 (32%)
It is discreet					
National schemes	19 (10%)	18 (9%)	43 (22%)	61 (31%)	57 (29%)
Local schemes	3 (5%)	6 (10%)	14 (22%)	20 (32%)	20 (32%)

We also compared respondents' views on the strengths and weaknesses of the four main types of system (stickers placed near the patient's bed and/or on patient notes; wristbands worn by the patient; electronic whiteboards and similar technologies; and summary bedside information documents) (Table 6) (Supplementary Material 2 Table 2). Some statistically significant differences in patterns of response by type of system were found.

**TABLE 6:** Responses to propositions regarding advantages and disadvantages of identifier systems, by system type

Proposition	Disagree	Some‐what disagree	Neutral	Some‐what agree	Agree
It is easy to notice					
Stickers	4 (3%)	11 (9%)	7 (6%)	34 (28%)	65 (54%)
Wristbands	0 (0%)	3 (11%)	3 (11%)	11 (41%)	10 (37%)
Whiteboards	2 (4%)	2 (4%)	7 (14%)	12 (24%)	27 (54%)
Bedside information documents	6 (9%)	12 (18%)	6 (9%)	19 (29%)	22 (34%)
It is easy to use					
Stickers	1 (1%)	2 (2%)	18 (15%)	25 (21%)	74 (62%)
Wristbands	0 (0%)	0 (0%)	2 (7%)	8 (29%)	18 (64%)
Whiteboards	2 (4%)	6 (12%)	5 (10%)	9 (18%)	28 (56%)
Bedside information documents	1 (2%)	6 (9%)	3 (5%)	20 (31%)	35 (54%)
It is helpful to staff					
Stickers	0 (0%)	3 (2%)	19 (16%)	28 (23%)	71 (59%)
Wristbands	1 (4%)	1 (4%)	4 (14%)	5 (18%)	17 (61%)
Whiteboards	2 (4%)	5 (10%)	7 (14%)	7 (14%)	29 (58%)
Bedside information documents	0 (0%)	1 (2%)	2 (3%)	18 (28%)	44 (68%)
Patients like it					
Stickers	1 (1%)	2 (2%)	75 (63%)	17 (14%)	25 (21%)
Wristbands	1 (4%)	2 (7%)	19 (68%)	4 (14%)	2 (7%)
Whiteboards	5 (10%)	2 (4%)	39 (78%)	2 (4%)	2 (4%)
Bedside information documents	1 (2%)	0 (0%)	29 (45%)	17 (26%)	18 (28%)
Carers like it					
Stickers	0 (0%)	1 (1%)	46 (38%)	34 (28%)	40 (33%)
Wristbands	1 (4%)	0 (0%)	15 (54%)	8 (29%)	4 (14%)
Whiteboards	5 (10%)	2 (4%)	37 (74%)	2 (4%)	4 (8%)
Bedside information documents	0 (0%)	0 (0%)	7 (11%)	24 (37%)	34 (52%)
No supply issues^a^					
Stickers	15 (13%)	21 (18%)	48 (40%)	21 (18%)	15 (13%)
Wristbands	5 (18%)	2 (7%)	6 (21%)	6 (21%)	9 (32%)
Whiteboards	7 (14%)	1 (2%)	13 (26%)	9 (18%)	20 (40%)
Bedside information documents	6 (9%)	7 (11%)	23 (35%)	11 (17%)	18 (28%)
It is used consistently					
Stickers	23 (19%)	22 (18%)	27 (22%)	33 (27%)	16 (13%)
Wristbands	8 (29%)	6 (21%)	3 (11%)	3 (11%)	8 (29%)
Whiteboards	5 (10%)	7 (14%)	11 (22%)	16 (32%)	11 (22%)
Bedside information documents	11 (17%)	19 (29%)	14 (22%)	14 (22%)	7 (11%)
It improves safety					
Stickers	7 (6%)	3 (3%)	37 (31%)	35 (29%)	38 (32%)
Wristbands	1 (4%)	2 (7%)	5 (18%)	10 (36%)	10 (36%)
Whiteboards	4 (8%)	2 (4%)	8 (16%)	17 (35%)	18 (37%)
Bedside information documents	0 (0%)	0 (0%)	14 (22%)	25 (38%)	26 (40%)
It is discreet					
Stickers	8 (7%)	11 (9%)	21 (18%)	43 (36%)	37 (31%)
Wristbands	4 (14%)	3 (11%)	6 (21%)	6 (21%)	9 (32%)
Whiteboards	7 (14%)	6 (12%)	13 (26%)	10 (20%)	14 (28%)
Bedside information documents	3 (5%)	4 (6%)	17 (27%)	22 (35%)	17 (27%)

a Phrasing of this proposition varied. For most identifiers, it was phrased: ‘We never run out of [identifier].’ For cut‐out symbols in wristbands, it was phrased: ‘It is easy to find the shape cutter when needed.’ For whiteboards, it was phrased: ‘Our electronic board has a special option for patients with dementia’.

First, there were significant differences in responses by type of identifier to questions regarding ease of use (*p* = .044) and helpfulness to staff (*p* = .028). Wristbands were viewed as easier to use than other systems; bedside leaflets were viewed as especially helpful to staff.

**FIGURE 4:**
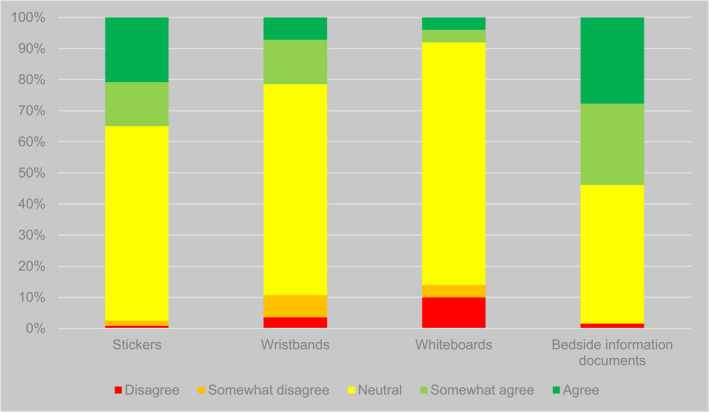
Responses to the proposition ‘Patients like it’ (percentages), disaggregated by type of system

**FIGURE 5:**
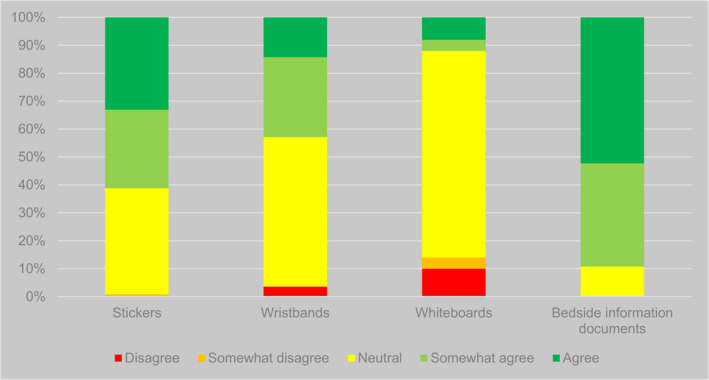
Responses to the proposition ‘Carers like it’ (percentages), disaggregated by type of system

Second, there were significant differences in participants' responses regarding the views of patients (*p* < .001) and carers (*p* < .001) on different types of system. Participants were more confident that patients and carers liked bedside documents (such as ‘This is me’ booklets) than other types of system. Over half of participants (35/65, 54%) agreed or somewhat agreed that patients liked such documents, and the vast majority (58/65, 89%) agreed or somewhat agreed that carers liked them. This contrasted with figures of 52/198 (26%) and 92/199 (46%), respectively, for all other types of identifier (Figures 4 and 5).

Positive views of bedside documents were also expressed in free‐text responses. Respondents gave many examples of using the information contained in such booklets to improve quality of care by, for example, de‐escalating confrontational situations or putting anxious patients at ease.

A well completed ‘This is Me’ can enable staff to discover likes and dislikes and this can improve many aspects of patient care, particularly as it focusses on the basics. (Manager)

However, some respondents also noted that completing these documents could be labour‐intensive, and that staff did not always have the time to read them and use them to inform their practice on busier days.

**FIGURE 6:**
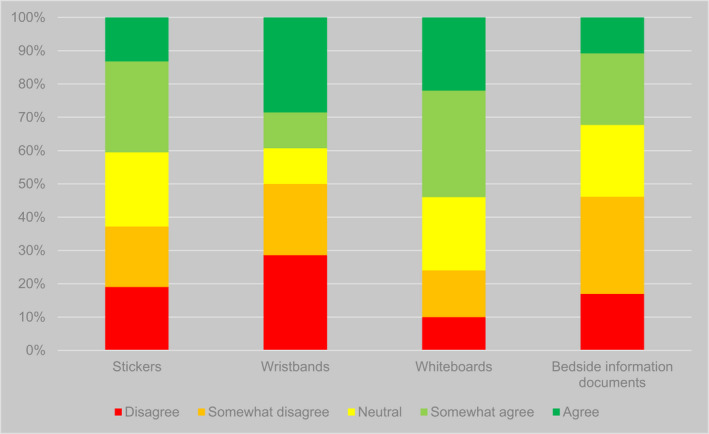
Responses to the proposition ‘It is used consistently’ (percentages), disaggregated by type of system

Third, there was variation in participants' views of the reliability and consistency of use of different types of identifier. There were significant differences in responses about supply issues by type of identifier (*p* = .010), with fewer participants indicating problems with whiteboard‐based systems than others (though this may be an artefact of the phrasing of the question for different types of identifier—see Table 6). Participants also indicated that electronic whiteboards were used more consistently for identifying patients with a diagnosis of dementia than other interventions, with 27/50 (54%) agreeing or somewhat agreeing that whiteboards were used consistently, compared with 81/214 (38%) agreeing or somewhat agreeing with the same proposition for other interventions (Figure 6); however, the difference between responses to this question did not reach statistical significance (*p* = .103).

Free‐text responses also highlighted the importance of ensuring that use of visual identifiers was complemented by staff training. Several responses highlighted poor training around appropriate care for patients living with dementia and argued for the importance of integrating high‐quality training into a wide range of hospital employees' roles. They made clear that the existence of a visual identification system was no substitute for training in dementia care; some suggested that the use of identifiers without training even risked having a negative effect on quality of care.

For a nurse like me, identifying dementia is another skill that we have to develop as part of our training and one should not resort to identifiable forms to be able to give them extra care. (Nurse)

I have worked for the trust for over 20 years and have never received any formal training. Substantive staff are sent on these courses as standard but bank staff are not. I strongly believe that all staff who have any direct patient contact (including porters/support staff should receive formal training and regular updates on how to care for our patients. It is not unusual for a member of staff to […] automatically assume the patient cannot communicate their needs and make certain decisions for themselves, or in some cases only talk to the member of staff involved in the patients care and ignore the patient. (Nursing/healthcare assistant)

## 4 DISCUSSION

Identification systems for dementia are in place in the vast majority of hospitals in England and Wales (Royal College of Psychiatrists, 2019), and their uptake has increased markedly over the last 10 years (Royal College of Psychiatrists, 2017, 2019). As in other areas of dementia care, however, the good intentions behind identification schemes might not be realised, or might even have negative unintended consequences (Featherstone et al., 2020; Featherstone & Northcott, 2020; Gwernan‐Jones et al., 2020), and they therefore warrant scrutiny. This survey provides some of the first insights into how the national recommendation to introduce such systems has been operationalised, identifying a range of schemes and systems in use and characterising staff perceptions of their strengths and weaknesses. Though staff views of visual identifiers for a diagnosis of dementia and other forms of cognitive impairment were broadly positive, they also highlight risks such as inconsistency, lack of staff training, uncertainty about patient and family views, and unclear consent processes. Participants' responses also make explicit some of the challenges, including those relating to the wider institutional determinants of quality of care.

One challenge relates to the need for a supportive infrastructure in high‐quality care for people with cognitive impairment (Murray et al., 2019), rather than seeing identifiers as a standalone intervention. This need is emphasised in resources used to support some of the national schemes, such as the Butterfly Scheme and Dementia Friendly. There is some evidence that identifiers deployed as part of a wider approach to dementia care, incorporating training and development for staff, can contribute to improvements in some aspects of care quality (Beattie et al., 2021; Murray et al., 2019). Conversely, a sizeable body of evidence points towards the problems of implementing dementia interventions in isolation, without attention to the wider institutional determinants of care quality, including availability of training (Featherstone et al., 2020; Featherstone & Northcott, 2020; Gwernan‐Jones et al., 2020). One possible risk is that visual identifiers might contribute to stigmatisation of hospitalised people with dementia, for instance by encouraging taken‐for‐granted assumptions about their condition and abilities instead of person‐centred care (Handley et al., 2017; Houghton et al., 2016).

One potentially useful approach would recognise visual identifiers as elements of socio‐technical systems (Holden et al., 2013), and accordingly as requiring purposeful work system design that accounts for training requirements and any additional organising work they may generate (Allen, 2014), including the impacts on the routines of already‐busy staff. More broadly, our study findings suggest some difficulties with consistent use of identifiers—perhaps reflecting the challenges faced in making reliable use of items that need to be gathered and used for each patient separately, as opposed to systems that are (literally) part of the furniture in a ward.

Future work should also address the challenges associated with the proliferation of different identifiers that we uncovered. We found multiple systems, used in various combinations. Some were established national schemes; some were adaptations of national schemes; and some were locally developed. The common use of multiple systems in combination may reflect the varying advantages and disadvantages of different types of system, as identified in our findings from both quantitative and free‐text analysis. By using different types of identifier system in combination, healthcare staff may be able to benefit from their respective advantages and mitigate their respective limitations.

Yet while tailoring and combining approaches may well have value, there is also the potential that the multiplicity of schemes—particularly ones with subtly different terminologies and expectations—may generate inconsistencies that are not only taxing for staff, but also introduce new risks (Dixon‐Woods, 2019), as well as potentially creating unnecessary waste. For instance, the excess of signs, symbols and artefacts is a potential threat to the economy of attention of the hospital ward, including the potential for confusion, and the loss of signals in the noise (Dixon‐Woods, 2019; Featherstone et al., 2020). Such concerns seem particularly pertinent to organisations where more than one type of identifier compete for attention. Having a multiplicity of systems also creates workload as staff move between or within settings, with their time spent in having to unlearn old systems and learn new ones (Dixon‐Woods & Martin, 2016). Once local proliferation of identifiers has occurred, however, it is far from straightforward to replace them with a single standard (Kriznik et al., 2019). Rather than a forlorn attempt to standardise, therefore, a better focus for improvement might be on optimising the ‘interoperability' of identifier systems: for example, generating frameworks that seek to align the use of stickers with the use of bedside information documents, and ensuring common understanding of how each is used for given patient groups in given situations. In the absence of such frameworks, however, a key challenge for nursing staff remains navigating the variety of systems in use, particularly when working across wards and organisations.

A further area of challenge arises in relation to patients and carers' engagement with the systems. With the exception of bedside information documents, staff were not confident that patients and carers liked the systems. Perhaps of concern, and in contradiction with the guidance supporting some schemes, the results suggested that decisions about whether to use an identifier were not always taken in discussion with patients and their informal carers. While the medico‐legal ramifications of ‘opt‐out’ approaches to use of identifiers are not entirely clear (given their ambiguous status in relation to the definition of ‘medical treatments’), from an ethical perspective there is an argument that identifiers that carry the risk of inadvertent disclosure of confidential information should be subject to an appropriate consent procedure (Brigden et al., 2020). Resolving some of these ethical dilemmas and developing new policies and processes to guide appropriate use of visual identifiers will be an important focus for future work for practitioners and researchers, and must be done collaboratively with patients and carers.

### 4.1 Strengths and weaknesses

Our study has strengths and weaknesses. A notable strength is that it is the first effort to systematically identify and obtain staff views on a class of interventions that has become commonplace in UK hospitals, but has to date (with a few exceptions [Featherstone et al., 2020]) evaded academic attention. It constitutes a first step towards understanding the broader implications of the use of visual identifiers in UK hospitals. As an exploratory survey, we were able to gain responses from staff in at least 48 organisations, but while the information about the survey was disseminated UK‐wide, most responses where the employing organisation was identified came from England. The findings therefore should not be taken to be statistically representative of national practice, and comparisons between views on different approaches should be interpreted with caution. A survey of this kind was not able to capture subtle differences in the way different types of identifier were used. We did not collect data on the type of setting in which participants worked (e.g. medical versus surgical wards), and the study suggests that more research is needed on how visual identification systems are used across different wards within hospitals or organisations. Finally, the survey covered healthcare staff only and is only one part of a programme of research that separately includes qualitative work on the views of people with dementia and their informal carers on visual identifiers and their use. This approach recognises that there is no substitute for research directly involving these groups, but also gives voice to staff experiences and perceptions of different systems that may be useful to address in the design and implementation of system.

### 4.2 Implications for Practice

This study highlights the importance of appropriate staff training and sound procedures to encourage good practice in contexts in which visual identifiers for patients with dementia are used. One important aspect of using visual identifiers for this population is consideration of the wishes of patients and their carers, including a clear consent process. In hospital environments in which multiple systems are in use, staff involved in care of people with dementia should be aware of the strengths and weaknesses of different systems, as well as potential challenges posed by use of multiple systems. Enhancing interoperability across units within the same hospital using different systems may be of benefit here. Another key issue is ensuring that identifiers and their implications for action are recognised by staff across all hospital areas, rather than being confined solely to wards where patients with dementia are typically cared for. Training may play a role in this, but so too may simpler interventions, for example awareness campaigns. Policy and practice in this area should focus on development of a sound evidence base that addresses effectiveness, cost‐effectiveness, work system design, advantages and disadvantages of standardisation, key features of training and other supporting infrastructure, patient and carer engagement, reduction of unnecessary waste, and ethics.

## 5 CONCLUSION

Though widely used in hospitals and other healthcare settings in the UK, visual identifier systems for patients with dementia and other forms of cognitive impairment are not free of challenge. Little is known about the effectiveness of the schemes, or about which scheme works best—and from whose perspectives. Our study indicates that multiple systems are in use in the UK, with healthcare settings often embracing more than one system. The survey also highlights the structural prerequisites of such systems, including adequate staffing to ensure they can be operated reliably and consistently, good work system design, and clarity about consent and other ethical concerns.

## CONFLICT OF INTEREST

The authors declare that they have no conflicts of interest.

## Supporting information


Supplementary Material 1

Supplementary Material 2

## Data Availability

The data that support the findings of this study are available from the corresponding author upon reasonable request.
