# Supplementary material for: Visual identifier systems for patients with cognitive impairment in healthcare settings: A survey of practice in UK hospitals
Source: Int J Older People Nurs. 2022 May 16;17(6):e12472. doi: 10.1111/opn.12472 (PMC9786885; doi:10.1111/opn.12472)
Supplement: Supplementary file 1 — Supplementary Material 1 [file OPN-17-e12472-s001.docx]

**Visual identifiers survey: Supplementary Material 1**

1. **Are visual identifiers used to recognise patients with dementia in your area of the hospital? Examples might include badges or wristbands worn by patients, or symbols near the patient’s bed or on electronic whiteboards.**
2. Yes
3. No
4. Not sure
5. Other [free text]
6. **Who chooses whether to use an identification symbol?**
7. Patients themselves opt in
8. Patients’ advocates (e.g. relatives or informal carers) opt in
9. Either patients or their advocates can opt in
10. Staff members decide whether to use it
11. Staff members decide only if patient lacks capacity and advocate is absent
12. Not sure
13. Other [free text]
14. **Do you use any of these visual identification symbols or systems for patients with dementia in your area of the hospital?**

**If a similar symbol or system is used in your workplace, please select “similar, not identical”. We will ask you about it a little later.**

**If no identical or similar symbol is used, go to the next row.**

| Online survey showed images of the following: | Yes | Similar, not identical | No |
| --- | --- | --- | --- |
| The Butterfly Scheme symbols |  |  |  |
| Forget-me-not Scheme symbol |  |  |  |
| Dementia Friendly symbol |  |  |  |
| The Purple Angel symbol |  |  |  |
| Blue hospital wristband photo |  |  |  |
| Digital hospital wristband photo |  |  |  |
| Flower-shaped cutout in hospital wristband |  |  |  |
| Electronic patient board photo |  |  |  |
| “This is me” leaflet image |  |  |  |

**[Question 4 was be repeated for every system identified by participants in response to Question 3]**

**In the next part of the survey we will ask your opinions of visual identifiers for patients with dementia used in your area of the hospital.**

1. **You have indicated that [visual identification system from Question 3] is used in your area of the hospital.**

**What do you like about this visual identifier or device?**

|  | Agree | Somewhat agree | Neither agree nor disagree | Somewhat disagree | Disagree |
| --- | --- | --- | --- | --- | --- |
| It is easy to notice |  |  |  |  |  |
| It is easy to use |  |  |  |  |  |
| It is helpful to staff |  |  |  |  |  |
| Patients like it |  |  |  |  |  |
| Carers like it |  |  |  |  |  |
| [As appropriate to the question]  We never run out of the stickers/ wristbands/leaflets/Our electronic board has a special option for patients with dementia |  |  |  |  |  |
| It is used consistently |  |  |  |  |  |
| It improves safety |  |  |  |  |  |
| It is discreet |  |  |  |  |  |

**[Questions 6–9 will be asked if participants select “similar, not identical” for the relevant visual identifiers shown in question 3]**

1. **We've shown you the following symbols used for patients with dementia:**

[images of: the Butterfly Scheme symbols, Forget-me-not, Dementia Friendly, and The Purple Angel]

**In your answer to question 3, you told us that you use similar symbols in your workplace. Please describe the symbol here:**

[free text]

**6a. What do you like about this symbol? [analogous to Question 4]**

**6b. Would you like to upload a photo of the symbol you have just told us about? Please make sure there are no patient faces, personal data, other identifying features) or hospital identifiers (such as hospital logos) in the photograph.**

1. Yes
2. No
3. **You told us that your area of the hospital uses a similar wristbands approach to identify patients with dementia:**

[images of: the blue hospital wristband, digital hospital wristband, a hospital wristband with a flower-shaped cutout]

**Please describe the approach used in your area of the hospital:**

[free text]

**[7a–7b analogous to 6a–6b]**

1. **You told us that your area of the hospital uses a similar patient board to identify people with dementia. Please tell us about it here:**

**[image of a digital hospital whiteboard]**

[free text]

**[8a–8b analogous to 6a–6b]**

1. **You told us that your area of the hospital uses a similar leaflet for patients with dementia. Please tell us about it here:**

[image of a “This is me” booklet]

[free text]

**[9a–9b analogous to 6a–6b]**

1. **Which of the options below best describes your role in the hospital?**
2. Nurse
3. Nursing assistant or healthcare assistant
4. Doctor or surgeon (including junior doctor)
5. Student doctor, student nurse or other clinical student
6. Physiotherapist
7. Occupational therapist
8. Pharmacist
9. Other allied health professional
10. Manager
11. Porter or domestic staff
12. Administrative staff
13. Other [free text]
14. **What is your job title?**

[free text]

1. **Which NHS organisation(s) are you employed at?**

[free text]

1. **Can you think of a situation where a visual identifier used for patients with dementia helped to deliver better care? If so, please describe it here.**

[free text]

1. **Can you think of a situation where a visual identifier used for patients with dementia could have resulted in worse-quality care? If so, please describe it here.**[free text]
2. **Is there anything else you would like to tell us about visual identification systems for patients with dementia? Please use the box below.**

[free text]
