# Supplementary material for: Visual identifier systems for patients with cognitive impairment in healthcare settings: A survey of practice in UK hospitals
Source: Int J Older People Nurs. 2022 May 16;17(6):e12472. doi: 10.1111/opn.12472 (PMC9786885; doi:10.1111/opn.12472)
Supplement: Supplementary file 2 — Supplementary Material 2 [file OPN-17-e12472-s002.docx]

**Visual identifiers survey – Supplementary Material 2: Statistical analyses**

| Proposition | Disagree | | Somewhat disagree | | Neutral | | Somewhat agree | | Agree | |
| --- | --- | --- | --- | --- | --- | --- | --- | --- | --- | --- |
|  | Observed | Expected | Observed | Expected | Observed | Expected | Observed | Expected | Observed | Expected |
| It is easy to notice |  | |  | |  | |  | |  | |
| National schemes | 10 | 9.2 | 24 | 21.4 | 15 | 17.6 | 60 | 58.1 | 92 | 94.8 |
| Local schemes | 2 | 2.8 | 4 | 6.6 | 8 | 5.4 | 16 | 17.9 | 32 | 29 |
| p=0.423 | | | | | | | | | | |
| It is easy to use |  | |  | |  | |  | |  | |
| National schemes | 3 | 3.0 | 13 | 10.6 | 24 | 21.3 | 49 | 47.1 | 111 | 117.9 |
| Local schemes | 1 | 1.0 | 3 | 3.4 | 4 | 6.7 | 13 | 14.9 | 44 | 37.1 |
| p=0.232 | | | | | | | | | | |
| It is helpful to staff |  | |  | |  | |  | |  | |
| National schemes | 2 | 2.3 | 8 | 7.6 | 28 | 24.4 | 46 | 44.2 | 117 | 122.6 |
| Local schemes | 1 | 0.7 | 2 | 2.4 | 4 | 7.6 | 12 | 13.8 | 44 | 38.4 |
| p=0.421 | | | | | | | | | | |
| Patients like it |  | |  | |  | |  | |  | |
| National schemes | 5 | 6.1 | 6 | 4.6 | 118 | 123.2 | 32 | 30.4 | 39 | 35.7 |
| Local schemes | 3 | 1.9 | 0 | 1.4 | 44 | 38.8 | 8 | 9.6 | 8 | 11.3 |
| p=0.268 | | | | | | | | | | |
| Carers like it |  | |  | |  | |  | |  | |
| National schemes | 4 | 4.6 | 3 | 2.3 | 83 | 79.9 | 42 | 51.8 | 69 | 62.4 |
| Local schemes | 2 | 1.4 | 0 | 0.7 | 22 | 25.1 | 26 | 16.2 | 13 | 19.6 |
| p=0.015 | | | | | | | | | | |
| No supply issues |  | |  | |  | |  | |  | |
| National schemes | 25 | 25.1 | 19 | 23.6 | 73 | 68.4 | 35 | 35.7 | 48 | 47.1 |
| Local schemes | 8 | 7.9 | 12 | 7.4 | 17 | 21.6 | 12 | 11.3 | 14 | 14.9 |
| p=0.277 | | | | | | | | | | |
| It is used consistently |  | |  | |  | |  | |  | |
| National schemes | 36 | 35.8 | 35 | 41.1 | 45 | 41.9 | 50 | 50.3 | 35 | 32.0 |
| Local schemes | 11 | 11.2 | 19 | 12.9 | 10 | 13.1 | 16 | 15.8 | 7 | 10.0 |
| p=0.200 | | | | | | | | | | |
| It improves safety |  | |  | |  | |  | |  | |
| National schemes | 12 | 9.1 | 5 | 5.3 | 49 | 48.6 | 61 | 66.1 | 72 | 69.9 |
| Local schemes | 0 | 2.9 | 2 | 1.7 | 15 | 15.4 | 26 | 20.9 | 20 | 22.1 |
| p=0.216 | | | | | | | | | | |
| It is discreet |  | |  | |  | |  | |  | |
| National schemes | 19 | 16.7 | 18 | 18.2 | 43 | 43.2 | 61 | 61.4 | 57 | 58.4 |
| Local schemes | 3 | 5.3 | 6 | 5.8 | 14 | 13.8 | 20 | 19.6 | 20 | 18.6 |
| p=0.827 | | | | | | | | | | |

**Table 1**. Responses to propositions regarding advantages and disadvantages of identifier systems, by established national versus locally developed schemes: chi-square test results.

| Proposition | Disagree | | Somewhat disagree | | Neutral | | Somewhat agree | | Agree | |
| --- | --- | --- | --- | --- | --- | --- | --- | --- | --- | --- |
|  | Observed | Expected | Observed | Expected | Observed | Expected | Observed | Expected | Observed | Expected |
| It is easy to notice |  | |  | |  | |  | |  | |
| Stickers | 4 | 5.5 | 11 | 12.9 | 7 | 10.6 | 34 | 35.0 | 65 | 57.0 |
| Wristbands | 0 | 1.2 | 3 | 2.9 | 3 | 2.4 | 11 | 7.8 | 10 | 12.7 |
| Whiteboards | 2 | 2.3 | 2 | 5.3 | 7 | 4.4 | 12 | 14.4 | 27 | 23.6 |
| Bedside documents | 6 | 3.0 | 12 | 6.9 | 6 | 5.7 | 19 | 18.8 | 22 | 30.6 |
| p=0.063 | | | | | | | | | | |
| It is easy to use |  | |  | |  | |  | |  | |
| Stickers | 1 | 1.8 | 2 | 6.4 | 18 | 12.8 | 25 | 28.3 | 74 | 70.7 |
| Wristbands | 0 | 0.4 | 0 | 1.5 | 2 | 3.0 | 8 | 6.6 | 18 | 16.5 |
| Whiteboards | 2 | 0.8 | 6 | 2.7 | 5 | 5.3 | 9 | 11.8 | 28 | 29.5 |
| Bedside documents | 1 | 1.0 | 6 | 3.5 | 3 | 6.9 | 20 | 15.3 | 35 | 38.3 |
| p=0.044 | | | | | | | | | | |
| It is helpful to staff |  | |  | |  | |  | |  | |
| Stickers | 0 | 1.4 | 3 | 4.6 | 19 | 14.7 | 28 | 26.6 | 71 | 73.8 |
| Wristbands | 1 | 0.3 | 1 | 1.1 | 4 | 3.4 | 5 | 6.2 | 17 | 17.1 |
| Whiteboards | 2 | 0.6 | 5 | 1.9 | 7 | 6.1 | 7 | 11.0 | 29 | 30.5 |
| Bedside documents | 0 | 0.7 | 1 | 2.5 | 2 | 7.9 | 18 | 14.3 | 44 | 39.6 |
| p=0.028 | | | | | | | | | | |
| Patients like it |  | |  | |  | |  | |  | |
| Stickers | 1 | 3.7 | 2 | 2.7 | 75 | 73.9 | 17 | 18.3 | 25 | 21.4 |
| Wristbands | 1 | 0.9 | 2 | 0.6 | 19 | 17.2 | 4 | 4.3 | 2 | 5.0 |
| Whiteboards | 5 | 1.5 | 2 | 1.1 | 39 | 30.8 | 2 | 7.6 | 2 | 8.9 |
| Bedside documents | 1 | 2.0 | 0 | 1.5 | 29 | 40.0 | 17 | 9.9 | 18 | 11.6 |
| p<0.001 | | | | | | | | | | |
| Carers like it |  | |  | |  | |  | |  | |
| Stickers | 0 | 2.8 | 1 | 1.4 | 46 | 48.1 | 34 | 31.2 | 40 | 37.6 |
| Wristbands | 1 | 0.6 | 0 | 0.3 | 15 | 1.1 | 8 | 7.2 | 4 | 8.7 |
| Whiteboards | 5 | 1.1 | 2 | 0.6 | 37 | 19.9 | 2 | 12.9 | 4 | 15.5 |
| Bedside documents | 0 | 1.5 | 0 | 0.7 | 7 | 25.9 | 24 | 16.7 | 34 | 20.2 |
| p<0.001 | | | | | | | | | | |
| No supply issues |  | |  | |  | |  | |  | |
| Stickers | 15 | 15.1 | 21 | 14.1 | 48 | 41.1 | 21 | 21.4 | 15 | 28.3 |
| Wristbands | 5 | 3.5 | 2 | 3.3 | 6 | 9.6 | 6 | 5.0 | 9 | 6.6 |
| Whiteboards | 7 | 6.3 | 1 | 5.9 | 13 | 17.1 | 9 | 8.9 | 20 | 11.8 |
| Bedside documents | 6 | 8.2 | 7 | 7.7 | 23 | 22.2 | 11 | 11.6 | 18 | 15.3 |
| p=0.010 | | | | | | | | | | |
| It is used consistently |  | |  | |  | |  | |  | |
| Stickers | 23 | 21.5 | 22 | 24.8 | 27 | 25.2 | 33 | 30.3 | 16 | 19.3 |
| Wristbands | 8 | 5.0 | 6 | 5.7 | 3 | 5.8 | 3 | 7.0 | 8 | 4.5 |
| Whiteboards | 5 | 8.9 | 7 | 10.2 | 11 | 10.4 | 16 | 12.5 | 11 | 8.0 |
| Bedside documents | 11 | 11.6 | 19 | 13.3 | 14 | 13.5 | 14 | 16.3 | 7 | 10.3 |
| p=0.103 | | | | | | | | | | |
| It improves safety |  | |  | |  | |  | |  | |
| Stickers | 7 | 5.5 | 3 | 3.2 | 37 | 29.3 | 35 | 39.8 | 38 | 42.1 |
| Wristbands | 1 | 1.3 | 2 | 0.7 | 5 | 6.8 | 10 | 9.3 | 10 | 9.8 |
| Whiteboards | 4 | 2.2 | 2 | 1.3 | 8 | 12.0 | 17 | 16.3 | 18 | 17.2 |
| Bedside documents | 0 | 3.0 | 0 | 1.7 | 14 | 15.9 | 25 | 21.6 | 26 | 22.8 |
| p=0.231 | | | | | | | | | | |
| It is discreet |  | |  | |  | |  | |  | |
| Stickers | 8 | 10.1 | 11 | 11.0 | 21 | 26.2 | 43 | 37.2 | 37 | 35.4 |
| Wristbands | 4 | 2.4 | 3 | 2.6 | 6 | 6.1 | 6 | 8.7 | 9 | 8.3 |
| Whiteboards | 7 | 4.2 | 6 | 4.6 | 13 | 10.9 | 10 | 15.5 | 14 | 14.8 |
| Bedside documents | 3 | 5.3 | 4 | 5.8 | 17 | 13.8 | 22 | 19.6 | 17 | 18.6 |
| p=0.447 | | | | | | | | | | |

**Table 2**. Responses to propositions regarding advantages and disadvantages of identifier systems, by system type: chi-square test results.
